# Multiple Fibrolipomas of the Tongue: A Rare Case Report of a Pediatric Patient With Whole Exome Sequencing of the C2CD3 Gene

**DOI:** 10.1155/crid/5923373

**Published:** 2024-12-19

**Authors:** Fouad Alomari, Zahra H. Al Zayer, Hanaa Mohammad Alferdous

**Affiliations:** ^1^Department of Maxillofacial Surgery and Diagnostic Sciences, King Faisal Medical City, Abha, Saudi Arabia; ^2^Dental Department Institute, Al-Ahsa Health Cluster, Ministry of Health, Abha, Saudi Arabia; ^3^Department of Maxillofacial Surgery and Diagnostic Sciences, College of Dentistry, King Khalid University, Abha, Saudi Arabia

**Keywords:** C2CD3 gene, multiple fibrolipomas of the tongue, pediatric patient, rare entity, whole exome sequencing

## Abstract

Multiple fibrolipomas of the tongue are rare benign tumors with a prevalence of 0.2% among both adults and children. Moreover, this lesion affecting an infant has not been reported in the literature. This is the first reported case of multiple fibrolipomas of the tongue in an infant. This case report describes the genetic sequencing and treatment of a 1-year-old child suffering from multiple fibrolipomas. Irregular growths on the anterior and lateral border of the tongue were reported by the mother of the child at the time of birth. The patient was presented to the hospital at the age of 1, and surgical excision of the lesions was performed under general anesthesia. The surgery was uneventful. Genetic sequencing was performed via whole exome sequencing, and two variants of the C2CD3 gene have been identified that may be associated with this condition, although causation has not yet been confirmed. Although this is a rare entity of the oral cavity, there are various differential diagnoses. Therefore, maxillofacial surgeons should perform histological diagnosis to confirm the findings. This is the first study in literature to understand the genetic sequencing of multiple fibrolipomas in an infant. Hence, the finding of this report can be utilized in further studies registering such cases.

## 1. Introduction

Lipoma is a common benign tumor of connective tissue origin that can occur at any site with or without the involvement of adipose tissue [1]. Oral fibrolipoma is a rare entity that can occur at any site of the oral cavity, including the lips, tongue, pharynx, palate, and floor of the mouth. The prevalence of lipoma of the tongue is 0.2% [2, 3], and in the literature, only one case of multiple spindle-cell lipomas of the tongue in a 7-year-old child has been reported [4]. This is the first case of multiple fibrolipomas of the tongue involving a child at 1 year of age.

Lipoma is a benign neoplasm involving adipose tissue and always appears as an intact capsule [1–3, 5]. Moreover, intramuscular lipomas, also known as infiltrating lipomas, are usually not intact to the capsule and possess irregular boundaries. However, when lipomas involve other forms of tissues (epithelial, connective, or nerve tissue), they are microscopically classified as angiolipoma, neurofibrolipoma, angiomyolipoma, or fibrolipoma. Different classifications of lipoma have also been reported in the literature: (1) familial lipoma, associated with genetic and neural tissue origins; (2) multiple painful lipomas; (3) diffuse lipoma of the neck and shoulder region mainly affecting elderly males; (4) retroperitoneal lipoma, an unusually large lipoma of abdominal tissue origin; (5) malignant liposarcoma, a larger tumor (more than 10 cm) with the capacity for metastasis; and (6) brown lipoma, involving red blood cells, also termed hibernoma [1–3, 5].

Lipomas can occur at any site in the body, but these tumors are rare in adipose tissue-free areas such as the tongue [6, 7]. Tongue lipomas are clinically diagnosed as round-lobulated or oval masses of submucosal and sublingual tissues that are slow-growing, painless, and asymptomatic lumps [6, 7]. Unless affected by trauma, infection, chronic irritation, or hormonal or metabolic abnormalities, these tumors have smooth surfaces without ulceration. Despite their rarity, tongue lipomas are usually benign and treatable. The diagnosis of this lesion can be confirmed by clinical and radiological imaging. Moreover, histopathological examination is important for confirming these findings.

The etiopathology of this tumor remains unclear; however, the involvement of endocrine, mechanical, and inflammatory cells has been reported [5, 8, 9]. The common etiology of lipoma of the tongue is an alteration in lipid metabolism and an anomalous deposition of adipose tissue in the tongue [5, 8, 9]. It is also suggested that mild trauma to the tongue that increases tissue proliferation may also lead to lipoma of the tongue. Chromosomal involvement has been reported in a few cases of lipoma in which chromosome 12q, 13q, and 3p rearrangements are observed [10, 11].

Treatment of this lesion usually involves surgical excision, and a postoperative histopathological diagnosis is required to confirm the findings. This case report describes a rare case of multiple tongue fibrolipomas in a 1-year-old child who underwent surgical management. This article will also emphasize the genetic understanding of fibrolipoma.

## 2. Case Presentation

A 1-year-old male patient with multiple fibrous growths on the anterior and lateral border of the tongue was reported at the maxillofacial department at a tertiary hospital in the Aseer region of Saudi Arabia on January 21, 2024. The patient's mother first reported swelling at the time of birth, and the swelling slowly increased. The patient reported no history of pain but was irritated due to the swelling. The patient had difficulties masticating. The patient was the result of a consanguineous marriage.

**Figure 1:**
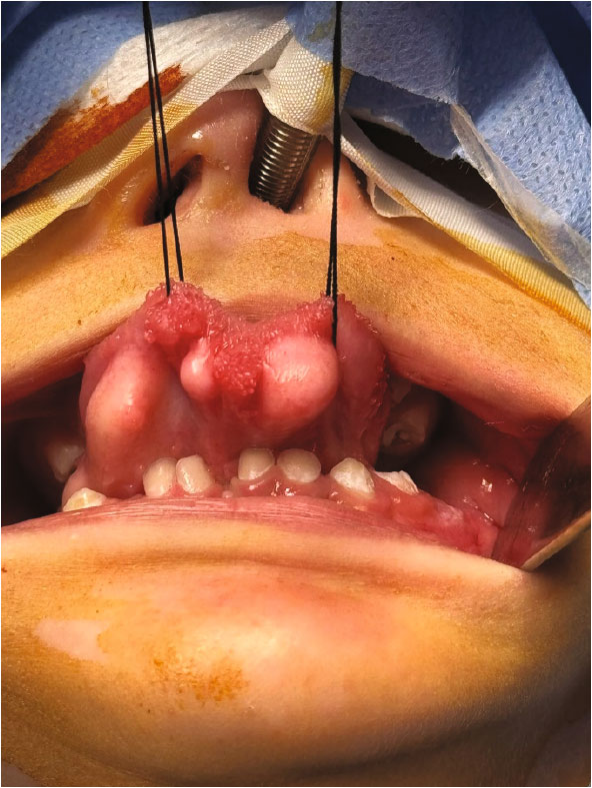
Multiple fibromatous growths of the tongue.

Intraoral examination revealed multiple tumors at the lateral and anterior border of the tongue, erupting molars, and early childhood caries affecting the maxillary anterior region. All the tumors were polypoid and covered under healthy mucosa ranging from 0.5 to 1.5 cm (Figure 1). It was doughy in consistency, not tender, and no pulsations were felt on palpation. Before confirming the lesion and diagnosis, tests like radiographic and histopathological examination were performed. Biopsy was not done on the patient due to the young age. Consultation from general physicians, pediatric surgeon, and ear, nose, and throat (ENT) specialists was done. The preliminary differential diagnosis was determined to be angioedema, neurofibroma, lipoma, fibrolipoma, glandular cell tumor, schwannoma, or dermoid cyst of the tongue. After clinical evaluation, radiographic findings, and histological examination, multiple tongue fibrolipomas were diagnosed.

**Figure 2:**
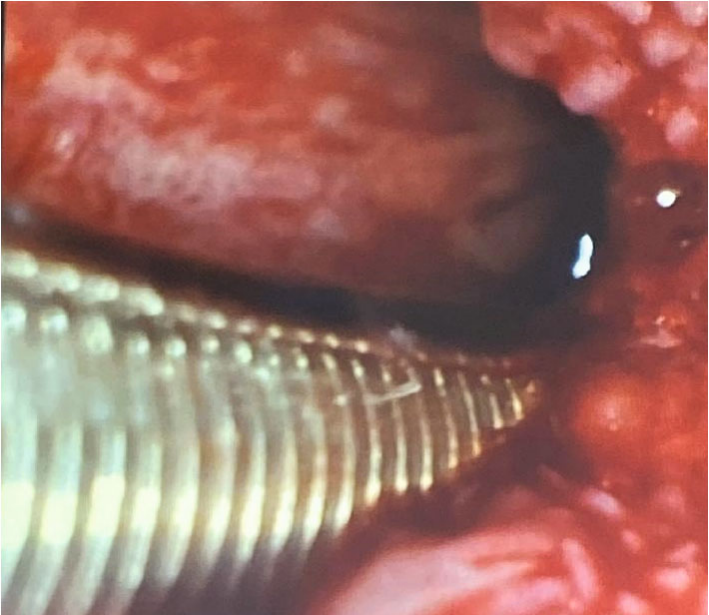
Fiberoptic imaging showing multiple granules in the pharynx.

Extraoral examination revealed no identified mass or swelling around the neck or head region. Submandibular and cervical lymphadenopathy was unremarkable. However, upon examination of the pharynx through fiberoptic imaging, multiple lipolytic granules were reported (Figure 2). These lesions were not painful. The patient was referred to the ENT department for the treatment of these lesions.

**Figure 3:**
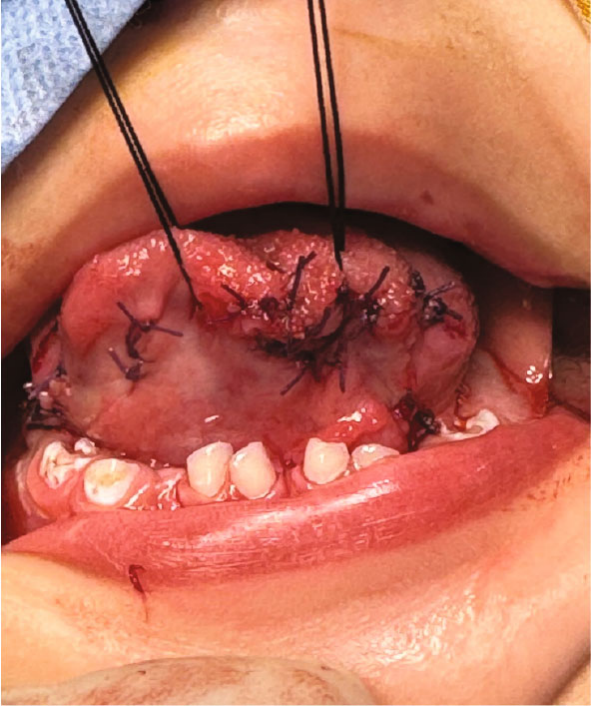
Multiple sutures given to the patient.

**Figure 4:**
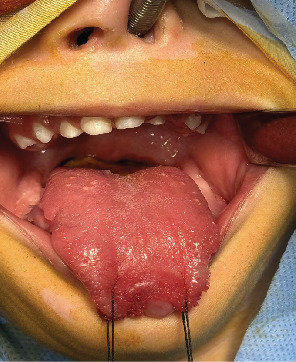
Healed tongue lesions after a week.

The treatment options were explained to the parents, and written consent was obtained. The surgery was performed under general anesthesia. The lesions were marked and, one by one, removed. Longitudinal incisions were created on the anterior and lateral sides of the affected tongue. Blunt dissections were made throughout the removal process, and the lesions were removed from the underlying mucosa. All the excised masses had slight yellowish tints and were well capsulated. En bloc excision was used to remove the lesions from the anterior and lateral surfaces. Following hemostasis, the defect areas were sutured using a resorbable suture (Figure 3). The postoperative instructions were explained to the parents, and the patient was kept on soft food till the healing was completed. The surgical site healed uneventfully (Figure 4).

**Figure 5:**
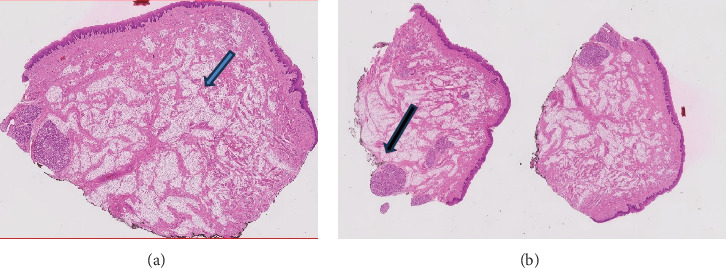
(a) Adipocytes in the center (blue arrow); (b) minor salivary gland foci at the border of the lesion (black arrow).

The excised masses were kept in formalin solution and sent to a laboratory for further investigation. Histopathological examination revealed adipocytes arranged in lobules and multiple erythrocytes (Figures 5(a) and 5(b)). Mild chronic inflammation and minor salivary gland foci were also reported.

## 3. Genetic Findings

As this was a rare case of tongue fibroma in a 1-year-old child, maxillofacial surgeons planned to perform genetic sequencing to evaluate whether the case was related to any orofacial developmental anomalies. Familial lipoma is said to be of genetic origin and affects family members throughout generations. In the current case, the patient's parents reported no such developments among any family members.

Genomic sequencing of the C2CD3 gene was performed to evaluate whether the developed lesion was congenital (Supporting Information (available here)). Most craniofacial anomalies are associated with mutations in C2CD3 genes in neural crest tissue. Pathologic variants of C2CD3 genes are reported to be causative factors for autonomic recessive orofaciodigital syndrome XIV. The characteristic features of this syndrome are abnormal facial growth, cleft lip, palate, and bifid tongue.

### 3.1. Whole Exome Sequencing (WES)

Genomic DNA was fragmented from the salivary sample of the child, and the corresponding exon–intron boundaries were enriched using Rocha KAPA technology (KAPA HyperExome library), amplified, and sequenced by Illumina technology (next-generation sequencing (NGS)). The target region was sequenced with an average coverage of 89.8-fold. The data were then aligned to the hg19 genome. Annotations were then performed using a bioinformatics pipeline. Identified variants and indels were filtered against external and internal databases and filtered depending on their allele frequency focusing on rare variants with a minor allele frequency (MAF) of 1% or less (Genome Aggregation Database (gnomAD) browser), and other homogenous variants were removed. The identified variants were classified according to American College of Medical Genetics and Genomics (ACMG) guidelines [12], which are considered database entries.

### 3.2. Results

Heterozygous sequencing of the C2CD3 gene was reported, and the c.503. C>T p.(PRO168) and c,133G>T. p(Val45Phe) was found to be linked to the amino acid exchange mechanism (Table 1). The sequence of this variant has not been documented among the general population. The armed forces laboratory in Saudi Arabia has claimed that this case is the first to be reported in their laboratory. Considering this information, the variant was classified as having uncertain significance. This finding supports the abnormality observed in the orofacial development of the patient.

When analyzing the genomic sequencing of both parents, it was found that C2CD3 genes were in heterozygous states. This finding infers that if both the parents had C2CD3 genes in heterozygous state and have a disorder, 75% of chances are there that their offspring will have genetic conditions related to the sequencing of the gene (Table 2). However, in the current case, none of the parents had any orofacial anomalies. The genetic report of both parents suggests genetic counselling for their offsprings.

## 4. Discussion

This is the first case in literature to report genomic sequencing of the C2CD3 gene in an oral lesion in a 1-year-old child. C2CD3 encodes a protein with multiple C2 domains, 5–6 at the carboxyl terminus and one novel, C2 domain at the amino terminus [13]. C2 domains have been shown to be involved in protein interactions with other ciliopathy proteins that localize to the transition zone, which lies distal to the basal body [13]. The WES results revealed that two variants (c.503C>Tp. (PRO168Leu) Chr11:72850854; c.133G>Tp. (Val45Phe) chr11:73879581) were responsible for the clinical condition of the patient. Both variants are of uncertain significance and have not been found in the literature, making this study the first of its kind. The variant (c.503C>Tp. PRO168Leu (Chr11:72850854)) is found in 0.0033% of cases worldwide [13], and the prevalence of the second variant is c.133G>Tp. (Val45Phe) chr11:73879581 is nil [13]. The C2CD3 gene is responsible for causing severe skeletal dysplasia; however, in the current case, maxillofacial surgeons did not record any skeletal anomalies.

The present patient had fibrolipoma of the tongue, and only three studies in the literature have reported the genetic sequence of the lesion [10, 11, 13]; this is the fourth study. These studies reported the translocation of chromosomes 12 and 16 [10, 11, 13]. In the study by Sreekantaiah, Leong, and Sandberg, the fibrolipoma displayed abnormalities in nine cells with chromosome 46, XY, inv(9)(pllq13), 11 cells with chromosome 46, XY, t(1;12)[p23;q14) inv(9)(pllq13), and five cells with chromosome 46, XY [10]. Another study described the involvement of only one chromosomal cell in the cases of fibrolipoma 46, XY, del(4)(q23), del(6)(q13), and inv(13)(q12q32) [11]. No specific anomalies that might distinguish fibrolipoma from classical lipomas have been reported. In the current case study, the patient was 1 year old; hence, genomic sequencing of C2CD3 was performed. This gene is thought to be responsible for congenital orofacial disorders.

In the literature, only four studies have reported lipomas in children involving the tongue [1, 7, 14–16]. The current reported case is an extremely rare case of tongue fibroma affecting a 1-year-old child. Its congenital origin was confirmed by the WES report of C2CD3 gene sequencing. The most common types of lipomas reported in children are familial, congenital, and present at birth. These lesions are poorly demarcated and increase with age, along with bone hypertrophy and venous angioma [2, 14]. In a case report of a 7-year-old child, spindle cell lipoma of the tongue was detected, where the lesion was a multilobulated and painless mass. In this case, CD34 expression was positive in the immunohistology report [4]. In a 4-year-old child in China, familial lipoma of the tongue was detected, and histological examination showed mature adipose tissue [6]. All the cases reported in literature have different histopathological findings which could be due to variation in the types of lipomas.

The differential diagnoses for a fibrolipoma of the tongue include mucocele, lymphangioma, hemangioma, neurofibroma, pleomorphic adenoma, salivary gland tumor, adenocarcinoma, and liposarcoma [17, 18]. In clinical practice, liposarcoma is frequently misdiagnosed as lipoma, even though liposarcoma can develop from a malignant change. Compared with other conditions, liposarcoma is more difficult to maintain and more strongly related to adjacent tissue [17, 18]. It is important to distinguish between lipoma of the tongue and liposarcoma.

Surgical excision of symptomatic or asymptomatic lesions is the treatment of choice for lipomas. Multiple lipomas are difficult to remove due to irregular boundaries. Similarly, it is not easy to complete en bloc resection of multiple lipomas, as the recurrence rate of multiple lipomas reported in the literature is approximately 65% [4, 16, 19]. In the present case, en bloc resection was planned due to the defined boundaries of the lesions. In this case, the surrounding normal tissue was also excised to prevent lesion recurrence.

## 5. Conclusion

The present case reported a rare case of multiple fibrolipomas of the tongue in a 1-year-old child. These lesions were removed by surgical excision. As the present case was reported in an infant, its congenital nature was evaluated via genomic sequencing of the C2CD3 gene via the WES technique. Two uncertain variants have been reported, making this case one of its kind. Future studies reporting on tongue fibrolipoma should consider the genomic findings of this study. Even though this is a rare entity of the oral cavity, there are various differential diagnoses. Therefore, maxillofacial surgeons should perform histological diagnosis to confirm the findings.

## Figures and Tables

**Table 1:** Results of whole exome sequencing of the patient.

**Gene (isoform)**	**Phenotype (mode of inheritance number)**	**Variant**	**Zygosity**	**MAF gnomAD (%)**	**Classification**
C2CD3	615948	c.503C>Tp.(PRO168Leu)Chr11:72850854	Heterozygous	0.0033	Uncertain significance
		c.133G>Tp.(Val45Phe)chr 11:73879581	Heterozygous	0	Uncertain significance

**Table 2:** Results of whole exome sequencing of parents.

**Gene (isoform)**	**Phenotype (mode of inheritance number)**	**Variant (mother)**	**Variant (father)**	**Zygosity**	**Classification**
C2CD3	NM_015531.6	Chr11:73879581 2:c.133G>Tp.Val45Phe	Chr11:73879581 2:c.133G>T, p.Val45Phe	Heterozygous	Uncertain significance
		Chr11:73850845 c.503C>T, p.Pro168Leu	Chr11:73850845 c.503C>T, p.Pro168Leu	Not detected	Uncertain significance

## Data Availability

Data will be provided upon request from the corresponding author.

## Supporting Information

Supporting Information Additional supporting information can be found online in the Supporting Information section. Genetic report of the child patient to understand the genomic sequencing of the affected C2CD3 gene of the patient.
