# Supplementary material for: Multiple Fibrolipomas of the Tongue: A Rare Case Report of a Pediatric Patient With Whole Exome Sequencing of the C2CD3 Gene
Source: Case Rep Dent. 2024 Dec 19;2024:5923373. doi: 10.1155/crid/5923373 (PMC11671624; doi:10.1155/crid/5923373)
Supplement: Supporting Information — Additional supporting information can be found online in the Supporting Information section. Genetic report of the child patient to understand the genomic sequencing of the affected C2CD3 gene of the patient. [file 5923373.f1.zip › 24S09150015-C2C3D Ex2 Mohammed (1).pdf]

GENALIVE
جينات الحياة

Sanger Sequencing-Based Genetic Testing Report

Patient Information
Name: Mohammed Ahmed Alahmari
Gender: Female
Date of birth: 24.11.1981
MRN/ ID: 1032217737

Sample Information
Sample ID: 24S09150015
Type: Periphral Blood
Date received: 15.9.2024
Date reported: 9.30.2024

Healthcare Provider Information
Referring Doctor: Dr.Fatima Asiri
Hospital/Institute: Specialized Medical Complex
Address: Abha - KSA

Test requested: Targeted testing for the below familial variants using Sanger sequencing:
- C2CD3, NM_015531.6, c.133G>T, p.Val45Phe
- C2CD3, NM_015531.6, c.503C>T, p.Pro168Leu

TEST CONCLUSION

The Targeted Familial Variant (p.Val45Phe) in the C2CD3 Gene Was Detected in a Heterozygous state
The Targeted Familial Variant (p.Pro168Leu) in the C2CD3 Gene Was Not Detected

TEST RESULTS

Gene, Transcript	Genomic Position (hg19)	Variants	Zygosity	OMIIM Phenotype(s) /Inheritance	Classification
**C2CD3,** NM_015531.6	Chr11:73879581	2:c.133G>T, p.Val45Phe	Heterozygous	Orofaciodigital syndrome XIV/ Autosomal recessive	Uncertain Significance
**C2CD3,** NM_015531.6	Chr11:73850845	c.503C>T, p.Pro168Leu	**Not Detected**		

RECOMMENDATIONS

- The clinical significance of the variant(s) described above should be interpreted in the context of the patient's phenotype and relevant family history.
- Genetic counseling is recommended.

METHODOLOGY AND LIMITATIONS

Methodology: DNA was extracted from the patient's blood or other tissues using standard protocols. Followed by the amplification of the target gene/region via PCR, using primers specific to the region of interest. The sequencing was conducted using an ABI genetic analyzer (Applied Biosystem) with BigDye terminator chemistry, and the chromatograms were generated and visualized using mutation surveyor software (SoftGenetics). The human reference genome hg19 was used as the reference genome.

Sanger sequencing limitations (include, but not limited to): the risk of allelic dropout, inability to detect complex or large deletion/duplication variants, and reduced sensitivity for detecting low-level mosaic variants and variants at repetitive or homopolymer regions.

Dr. Yusra Ahmed, PhD
Clinical Scientist

Dr. Salem Alawbathani, MD, PhD, ECMGG
Head of Molecular Genetics

Dr Fatima Asiri
Genetics
