# Supplementary material for: Multiple Fibrolipomas of the Tongue: A Rare Case Report of a Pediatric Patient With Whole Exome Sequencing of the C2CD3 Gene
Source: Case Rep Dent. 2024 Dec 19;2024:5923373. doi: 10.1155/crid/5923373 (PMC11671624; doi:10.1155/crid/5923373)
Supplement: Supporting Information — Additional supporting information can be found online in the Supporting Information section. Genetic report of the child patient to understand the genomic sequencing of the affected C2CD3 gene of the patient. [file 5923373.f1.zip › Fahad Mohd Ahmed.pdf]

**Name**	**: Fahad Mohd Ahmed**	**Sample Date**	**: 21/02/2024 12:59 PM**
**Lab. No.**	**: 67249049**	**Report Date**	**: 27/02/2024 11:45 AM**
**Contract.**	**: General network of health provider (Tadawi – GNP)**		
**Patient No.**	**: 5793-0341602**		
**File No.**	**:**		

**Branch : Khamis Mushait 2**	**Age : 2 Year**	**DOB : 21/02/2022**	**Sex : Male**	**Int. No. : 1S24.4379**

Pathology Unit

SP. Collection Date: **20/02/2024**

Biopsy (Small)

SPECIMEN:
Tongue lesion.

CLINICAL DATA:
Multi mucosal with mucous membrane from the lateral side of the tongue, soft with slightly epithelial tissue.
Primary diagnosis : Epithelial mucous lesion.

MACROSCOPY:
Received in a labelled container with patient identification and fixed in formalin, were multiple fragments of grayish soft tissue measuring 1 x 1 x 0.2 cm with separate ellipse of skin measuring 1 x 1 cm, inner surface inked black. All embedded in 2 cassettes as follows :
A = Multiple fragments
B = Ellipse of skin
Prepared by : Omnia Hamed on 22/02/2024

MICROSCOPY:
The examined tissue shows presence of a benign fibrolipomatous polyp (fibrolipoma) covered by non-keratinizing squamous epithelium with stroma of lobules of fat and fibrocollagenous septa. Also present at the periphery are foci of minor salivary glands and mild chronic inflammation. Negative for atypia or malignancy.

DIAGNOSIS:
Tongue lesion :
- Benign fibrolipomatous polyp (fibrolipoma).
- Negative for atypia or malignancy.

All Rights Reseved © National Technology
Powered by LDM www.nt-me.com

**Name**	**: Fahad Mohd Ahmed**	**Sample Date**	**: 21/02/2024 12:59 PM**
**Lab. No.**	**: 67249049**	**Report Date**	**: 27/02/2024 11:45 AM**
**Contract.**	**: General network of health provider (Tadawi – GNP)**		
**Patient No.**	**: 5793-0341602**		
**File No.**	**:**		

**Branch : Khamis Mushait 2**	**Age : 2 Year**	**DOB : 21/02/2022**	**Sex : Male**	**Int. No. : 1S24.4379**

Pathology Unit

SP. Collection Date: **20/02/2024**

Biopsy (Small)

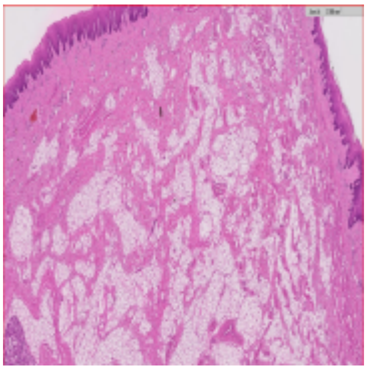

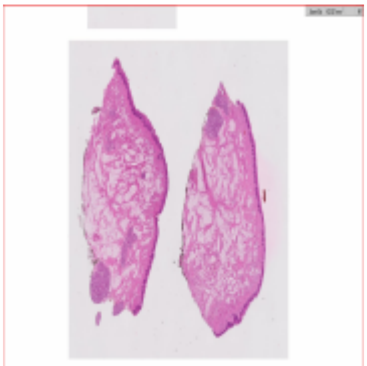

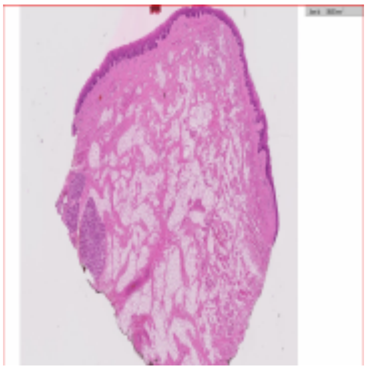

Verified By :

Dr. Ali Ahmed Eltayeb
MBBS, DCP, FRC path
Consultant Histopathologist
Head of Anatomic Pathology

All Rights Reseved © National Technology
Powered by LDM www.nt-me.com
